# RNAi-based screen for pigmentation in *Drosophila melanogaster* reveals regulators of brain dopamine and sleep

**DOI:** 10.1101/2023.07.20.549932

**Published:** 2025-04-02

**Authors:** Samantha L Deal, Danqing Bei, Shelley B Gibson, Harim Delgado-Seo, Yoko Fujita, Kyla Wilwayco, Elaine S Seto, Shinya Yamamoto

## Abstract

The dopaminergic system has been extensively studied for its role in behavior in animals as well as human neuropsychiatric and neurological diseases. However, we still know little about how dopamine levels are tightly regulated *in vivo* . To identify novel regulators of dopamine, we utilized *Drosophila melanogaster* cuticle pigmentation as a readout, where dopamine is a precursor to melanin. We measured dopamine from genes known to be critical for cuticle pigmentation and performed an RNAi-based screen to identify new regulators of pigmentation. We found 153 potential pigmentation genes, which were enriched for conserved homologs and disease- associated genes as well as developmental signaling pathways and mitochondria-associated proteins. From 35 prioritized candidates, we found 10 caused significant reduction in head dopamine levels and one caused an increase. Two genes, *clueless* and *mask (multiple ankyrin repeats single KH domain),* upon knockdown, reduced dopamine levels in the brain. Further examination suggests that Mask regulates the transcription of the rate-limiting dopamine synthesis enzyme, *tyrosine hydroxylase* , and its knockdown causes dopamine-dependent sleep phenotypes. In summary, by studying genes that affect cuticle pigmentation, a phenotype seemingly unrelated to the nervous system, we were able to identify several genes that affect dopamine metabolism as well as a novel regulator of behavior.

## Introduction

Dopamine is a conserved neurotransmitter that functions in the nervous system to regulate a variety of behaviors, including learning & memory, locomotion, reward, and sleep. In humans, disruptions in dopamine have been associated with neurological and neuropsychiatric disorders, including addiction, depression, sleep disorders, and schizophrenia(1–3). In addition, one of the hallmarks of Parkinson's disease is early degeneration of dopaminergic neurons, and Parkinson's patients are often given a dopamine precursor, l-dihydroxyphenylalanine (L-DOPA), as a method for treating symptoms (4,5). Most genetic research on dopamine biology has focused on dopaminergic signaling and transport; however, changes in dopamine levels can have a great impact on behavioral outcomes (6–8). Thus, identifying genes that affect dopamine levels is critical to understanding and potentially treating dopamine-associated disorders.

Dopamine synthesis is a conserved process, whereby the amino acid tyrosine is converted into L-DOPA via the rate-limiting enzyme, Tyrosine hydroxylase (TH) (9). Then, L-DOPA is converted into dopamine via the enzyme Dopa decarboxylase (Ddc) (10). In mammals, dopamine is degraded via oxidation or methylation using the enzymes Monoamine oxidase (MAO) and Catechol-O-methyltransferase (COMT), respectively (11). In invertebrates, dopamine degradation goes through β-alanylation via the β-alanyl amine synthase (encoded by the *ebony* gene in *Drosophila melanogaster*) and acetylation via the acetyltransferase (encoded by the *speck* gene in *Drosophila*) (12). However, insects also convert dopamine into melanin to form proper cuticle pigment and structure. In fact, changes in genes that are involved in dopamine metabolism also change the cuticle color (13–15). In other words, knockdown of *TH* (or the *pale (ple)* in *Drosophila*) or *Ddc* leads to a paler cuticle, and mutations or knockdown of *ebony* or *speck* leads to a darker cuticle.

Historically, *Drosophila* has been used to study dopamine biology in multiple contexts, including learning and memory (16,17). Over the years, an extensive genetic toolkit has been generated in the fruit fly, making it a great system for dissecting dopaminergic neural circuitry and signaling (18–20). In addition to the fly's contribution to understanding dopaminergic circuitry, this model organism helped to identify and investigate the cellular functions of Parkinson disease's genetic risk factors, *PINK1* and *PRKN* (i.e. *Pink1* and *parkin* in the fruit fly) (21). The conservation of systems in neural biology along with the efficiency of working in *Drosophila* has made it a great system for large-scale screening of novel genes. Previously, a large-scale RNAi screen (11,619 genes, ~89% of genome) was performed to identify genes that affect mechanosensory organs and cuticle formation in the *Drosophila* thorax (22). In this screen, the researchers found 458 genes that affected cuticle color upon knockdown, but no further validation was performed.

Here, we utilize the fruit fly cuticle to identify novel regulators of dopamine. We start by systematically examining classical pigmentation genes for their effect on dopamine in the head and brain. From this we found that genes that are involved in dopamine synthesis are the only genes with expected effects on dopamine; genes involved in degradation of dopamine either have no effect or unexpectedly show reduced dopamine. We go on to characterize 458 genes identified from the RNAi screen by Mummery-Widmer et al. (2009). We tested 330 of them with independent RNAi lines and found 153 genes (~46%) with consistent cuticle pigmentation defects. We examine 34 prioritized genes hits for changes in dopamine in the head and brain. From this analysis, we found two genes, *mask* and *clu*, that reduced brain dopamine levels upon knockdown in dopaminergic neurons. Lastly, we used molecular biology and sleep behavioral studies to show that *mask* appears to alter brain dopamine through regulation of TH transcription.

## Results

### *Drosophila* pigmentation genes affect dopamine in unexpected ways

To systematically test if changes in pigmentation genes affect dopamine as theorized in the field, we characterized loss-of-function alleles of genes involved in dopamine metabolism (Fig 1A) for pigmentation phenotypes and dopamine levels in the head and brain. The enzymes required for dopamine synthesis, TH and Ddc, are essential for survival (23,24). Thus, we took advantage of established RNAi lines and UAS/GAL4 system (25–28). It is known that reduction of *TH* or *Ddc* level or activity causes pale pigmentation and reduced dopamine (9,10,14). We validated our method by knocking down *TH* using a verified UAS-RNAi (29), which causes a strong pale cuticle phenotype (Figs 1B and 1-C), reduction in head dopamine by ~60% (Fig 1E), and reduction in brain dopamine by ~32% (Fig 1E’). *Ddc* knockdown causes a weaker effect than *TH* RNAi in the cuticle (Figs 1B and 1,D) and in the head (~35% dopamine reduction, Fig 1E), with no significant effect on dopamine in the brain (Fig 1E’). This may be due to the strength of the RNAi and/or enzyme level requirements (since TH is the rate-limiting enzyme).

103 **Figure 1**

104

**Fig 1. Manipulation of pigmentation enzymes have unexpected effects on dopamine.** (A) *Drosophila* dopamine metabolism pathway. Gene names encoding pigmentation enzymes are in blue and metabolites are in black. (B-D) Thorax phenotypes observed upon knockdown of Tyrosine hydroxylase (TH, also known as Pale) (C) and Dopa decarboxylase (Ddc) (D) compared to control (B). (E-E') Dopamine levels from the head (E) and brain (E') upon knockdown of *TH* and *Ddc*. Pigmentation phenotypes observed mutant alleles in the thorax (F-L) and the body (F'-L') (Arrow in I' indicates the darker region characteristic of *speck* mutants). *T2A-GAL4* alleles for *black*, *speck*, and *tan* adult brain expression with dopaminergic neurons (DAN) staining in magenta (DANs) (M-P). (Q) Dopamine in the head of classical pigmentation mutants, $black^1$, $ebony^1$, $straw^1$, and $yellow^1$. (R) Dopamine levels in dissected brains of classical pigmentation mutants. Ordinary one-way ANOVA test with Dunlett multiple comparisons test. *=p<0.05, **=p<0.01, ***=p<0.001, ****=p<0.0001. Error bars show standard deviation (SD).

To test genes implicated in dopamine degradation and cuticle development (pigmentation and structural rigidity), we examined known mutant alleles. These alleles cause darker or paler cuticles (Figs 1F-1L and, 1F'-1L') depending on their role in melanin metabolism. Unexpectedly, *black*, *speck*, *straw* and *yellow* mutants reduce head dopamine levels (Fig 1Q), while *ebony* and *tan* mutants had no significant effect (Fig 1Q). Next, we checked the brain expression patterns of pigmentation enzymes. Based on our own expression analysis (Figs 1M-1P) and previously reported expression data (30,31), most are expressed in the fly brain, but their expression does not appear to overlap with DANs. However, we found no significant effect on brain dopamine in these pigmentation genes (Fig 1R).

Together this data shows that changes in dopamine synthesis cause a paler cuticle, which reflects a reduction in dopamine, but changes in dopamine metabolism enzymes downstream of dopamine synthesis have pigmentation phenotypes that do not correlate with dopamine level differences.

### RNAi-based cuticle pigmentation screen

Mining a previously published genome-wide scale RNAi screen (22), we identified 458 genes with pigmentation defects. To check if these phenotypes can be reproduced, we located 718 additional UAS-RNAi lines corresponding to 330 genes. 426 lines came from the National Institute of Genetics in Mishima, Japan (32), and 292 came from the Transgenic RNAi Project (TRiP) at Harvard Medical School (25). 153 genes caused pigmentation defects in at least one additional RNAi line, showing a validation of 46.4% from the previous screen (Fig 2A).

## Figure 2

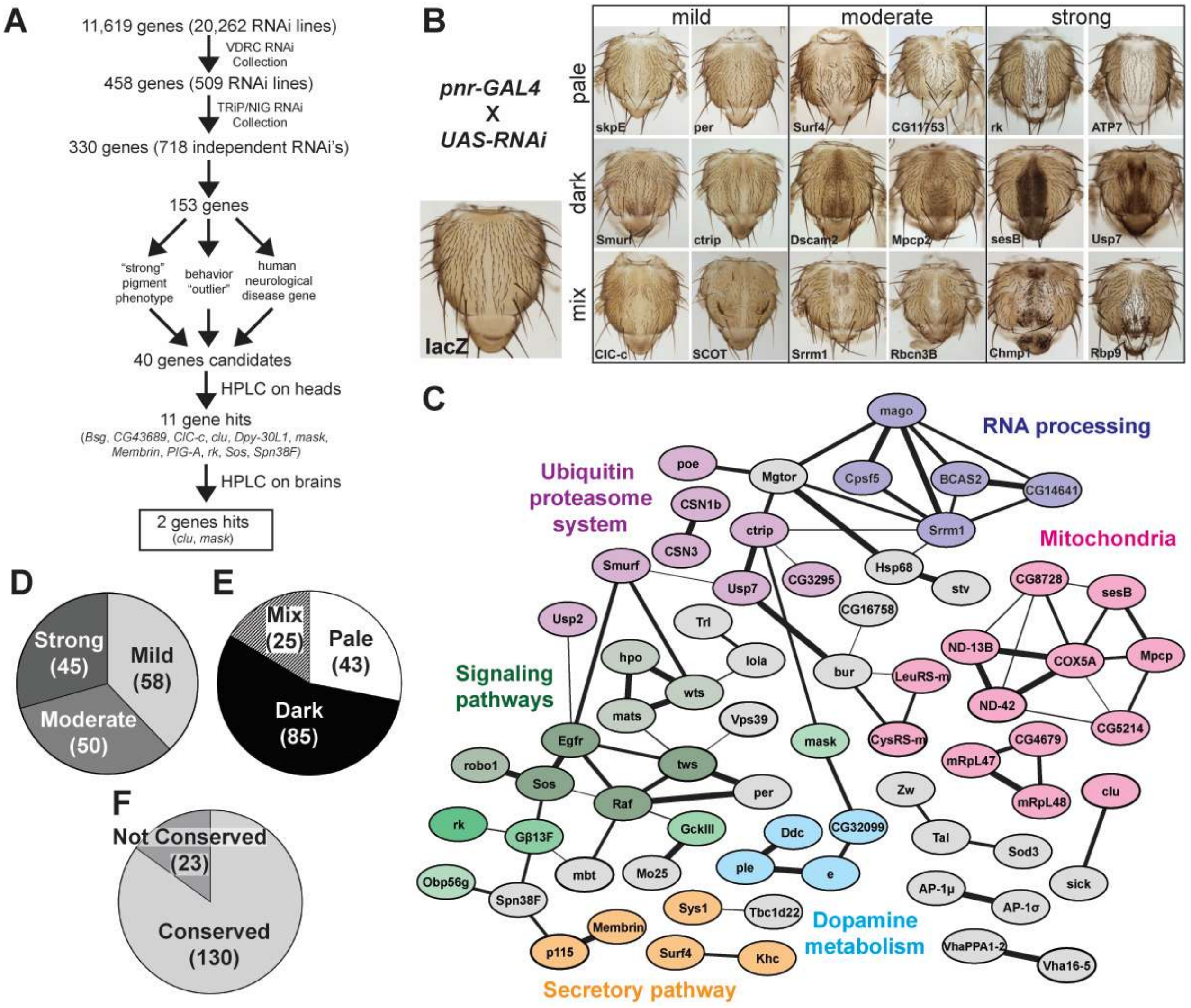

**Fig 2. RNAi pigmentation screen reveals a broad spectrum of phenotypes and classes of genes** (A) Flow chart depicting screening process. (B) Examples of the phenotypes observed from the RNAi-based pigmentation screen. (C) Protein-protein interaction network for 153 high confidence pigmentation gene hits from the RNAi-screen using STRING network analysis. Strength of line indicates confidence or strength of data support. Genes with no known interactions are not displayed. (D-F) Pie charts for the 153 pigmentation gene hits, including strength of phenotype (D), phenotype color (E), and conservation (F).

Our RNAi-based secondary screen produced a spectrum of phenotypes (Fig 2B), but the phenotypes strengths (mild, moderate, or strong) were equally represented (mild = 38%, moderate = 33%, strong = 29%, Fig 2D). Pigmentation color changes were classified in three primary categories, pale, dark, or mix (a combination of pale and dark in a single thorax), which were not equally represented (Fig 2E). Dark cuticle phenotypes were the most common (56%), with pale and mixed cuticle phenotypes seen in 28% and 16% of genes, respectively.

We performed a protein-network analysis (Fig 2C) and found a group of proteins previously linked to cuticle pigmentation as well as clusters related to RNA processing, ubiquitin proteasome system, mitochondria-associated proteins, and signaling pathway genes, Hippo and EGF (epidermal growth factor) signaling in particular. GO enrichment analysis highlighted terms associated with cuticle pigmentation, protein stability, and several signaling pathways (S1 Fig). We also observed that 85% of our genes were conserved between *Drosophila* and human, an enrichment from the 68% of conserved genes in the original screen (Fig 2F and S2 Fig).

In summary, the cuticle pigmentation screen identified a spectrum of pale, dark and mixed phenotypes in 153 genes, showing a validation of ~50% from the original screen. Close examination of these hits highlights conserved molecular processes.

### Many pigmentation genes isolated have a neurological consequence

To investigate if our gene candidates have a neurological consequence, we first checked if their homologs are associated with a neurological disease. We found that 51.2% of the fly gene homologs are associated with a disease, 78% of those have at least one disease with neurological phenotypes (Table 1 and S2 Fig), such as intellectual disability, autism spectrum disorder, ataxia, epilepsy, and cerebral atrophy.

**Table 1. Fly pigmentation genes identified from this screen with neurological disease associated homologs in human.**

| Fly Gene | Human Homologs with Neurological Phenotypes (DIOPT Score, Max=18) | Notable Neurological Phenotypes |
|---|---|---|
| AP-1sigma | AP1S2 (12), AP1S1 (11), AP2S1 (3) | ataxia, DD, seizures |
| ATP7 | ATP7A (13), ATP7B (13) | dystonia, neurodegeneration, seizures, tremor |
| Atu | LEO1 (10) | ASD |
| byn | TBXT (11), TBX19 (10) | neural tube defects, seizures |
| CG16758 | PNP (14) | ataxia, behavioral problems, spastic diplegia, tremor |
| CG4328 | LMX1B (10) | spina bifida |
| CG43689 | MYT1L (7) | DD, ID |
| CG4679 | PTCD3 (14) | cerebral atrophy, myoclonus, psychomotor delay |
| CG5902 | AAMERCR1 (13) | cerebral atrophy, early motor delay, myoclonus |
| CG8728 | PMPCA (15) | ataxia |
| ClC-c | CLCN3 (12), CLCN4 (11), CLCNKA (3) | ID, language delay, seizures, structural abnormalities |
| ctrip | TRIP12 (10) | delayed speech, ID, psychomotor delay |
| Cyp301a1 | CYP24A1 (5), CYP27B1 (4), CYP27A1 (4) | ataxia, motor delay, psychiatric symptoms, seizures |
| CysRS-m | CARS2 (15) | cerebral atrophy, epilepsy, psychomotor delay |
| Ddc | DDC (14), HDC (4) | dystonia, psychomotor delay, sleep disturbance |
| Dgp-1 | GTPBP2 (3) | ataxia, dystonia, ID, psychomotor delay, seizures |
| Dscam2 | DSCAM (10) | ASD |
| Egfr | ERBB4 (10), ERBB2 (10), ERBB3 (9) | ALS, axonal neuropathy, DD, seizures |
| Gbeta13F | GNB1 (15), GNB4 (13), GNB2 (12), GNB5 (3) | ADHD, CMT, DD, ID, language delay |

| | | |
|---|---|---|
| gig | TSC2 (13) | brain structural abnormalities, seizures |
| Hrb98DE | HNRNPA2B1 (12), HNRNPA1 (9) | ALS, dementia, motor neuropathy |
| Itpr | ITPR1 (14), ITPR3 (11) | ataxia, cerebellar atrophy, motor delay |
| Khc | KIF5C (12), KIF5A (12) | ALS, psychomotor delay, seizures, spastic paraplegia |
| Larp7 | LARP7 (10) | anxiety, DD, ID, unstable gait |
| LeuRS-m | LARS2 (15) | cognitive impairment, DD, dystonia, tremor, seizures |
| mask | ANKRD17 (10) | DD, ID, motor delay, speech delay |
| mbt | PAK1 (3), PAK3 (3), PAK2 (3) | ID, macrocephaly, seizures, speech delay |
| Membrin | GOSR2 (13) | cerebral atrophy, DD, seizures |
| Mp | COL18A1 (6) | ataxia, seizures |
| Mpcp2 | SLC25A3 (14) | motor delay |
| ND-13B | NDUFA5 (13) | ASD |
| ND-42 | NDUFA10 (14) | DD, strutural abnormalities |
| Npc2b | NPC2 (7) | behavioral problems, DD, dementia, dystonia, seizures |
| per | PER3 (8), PER2 (7) | sleep abnormalities |
| PIG-A | PIGA (15) | seizures, structural abnormalities |
| ple | TH (15) | ataxia, dystonia, Parkinsonism, motor and sleep delay |
| Prosap | SHANK2 (10), SHANK3 (8) | ASD, DD, schizophrenia, seizures |
| Raf | BRAF (14), RAF1 (11) | DD, seizures |
| Rbp9 | ELAVL2 (12), ELAVL3 (10) | ASD |
| rdx | SPOP (10) | DD, ID, speech delay |
| robo1 | ROBO3 (8) | structural abnormalities |

| | | |
|---|---|---|
| SCOT | OXCT1 (15) | coma, psychomotor delay |
| sesB | SLC25A4 (12) | motor delay, hyperreflexia |
| slp1 | FOXG1 (6), FOXI1 (3), FOXJ1 (3), FOXC1 (3) | dystonia, motor delay, sleep and structural abnormalities |
| Smurf | ITCH (4), NEDD4L (3) | ID, psychomotor delay, seizures |
| Sod3 | SOD1 (4) | ALS, spastic tetraplegia |
| Sos | SOS1 (14), SOS2 (12) | cognitive impair, learning disabilities |
| Spn38F | SERPINC1 (4), SERPINI1 (4) | cerebral atrophy, dementia, seizures |
| stv | BAG3 (9) | abnormal gait, behavioral problems, neuropathy |
| tws | PPP2R2A (13) | ataxia |
| Usp2 | USP8 (4) | behavioral problems |
| Usp7 | USP7 (13) | ID, psychomotor delay, seizures, structural abnormalities |

**Table 1.** Homologs were defined as a Drosophila Integrative Orthology Prediction Tool (DIOPT, version 8.0) score greater than or equal to three. Neurological disease associations were based on Online Mendelian Inheritance in Man (OMIM)_clincal disease phenotypes and high-confident ASD genes listed in Simons Foundation Autism Research Initiative (SFARI) gene database (class S, 1, 2 or 3). ASD=Autism Spectrum Disorder, DD=Developmental Delay, ID=Intellectual Disability, ALS=Amytrophic Lateral Sclerosis, CMT=Charcot-Marie-Tooth disease.

Since dopamine is known to regulate locomotion and sleep (22,33), we screened for locomotion and sleep defects using the Drosophila Activity Monitor (DAM) (34). We tested 274 RNAi lines from the NIG, TRiP, and VDRC RNAi collections (35–37) corresponding to the validated 152 gene hits. We scored the lines for total locomotion, total sleep, sleep bout length, and sleep latency during the night and day (Fig 3). There was a broad range in the RNAi lines for each of these phenotypes (Figs 3C). We classified RNAi lines “outliers” if they were two standard deviations from the mean for a given phenotype (locomotion: 23 lines, total sleep: 20 lines, sleep bout length: 21 lines, sleep latency: 25 lines). While total sleep and locomotion strongly overlapped with each other, sleep latency and sleep bout length did not overlap with the other sleep phenotypes (Fig 3D). We assessed if certain gene categories (>10 genes) showed any distinct behavioral patterns (Fig 2C). There was no change in total locomotion or total sleep in the categories assessed (Fig 3F and 3G). However, each categories showed a distinct pattern in sleep bout length and latency (Fig 3F and 3H). This could indicate a distinction in how different gene functions could affect sleep. Since genes that affect sleep can often show distinct phenotypes that do not necessarily correspond to multiple phenotypes or times of day (38), any gene with one phenotype during the day or night was classified as an “outlier”. This produced sixty lines, corresponding to 50 genes.

## Figure 3

**Fig 3. *Drosophila* Activity Monitor (DAM) screen identifies behavior "outliers" and highlights distinct phenotypes for gene categories.** (A-B) Representative activity and sleep data measured in a neutral RNAi line (*mCherry RNAi*, A) and a behavioral "outlier" (*Raf RNAi*, B) knocked down in DANs (*TH-GAL4*). (C) Bar graph showing all RNAi lines tested for 24hr locomotion and total sleep during the 12hr light period, where light pink/blue represents fly lines ±1SD away from the mean, and dark pink/blue are fly lines ±2SDs away from the mean (a.k.a. "outliers"). (D) Venn diagram of "outliers" for the four phenotypes examined (sleep latency, sleep bout length, locomotion, and total sleep). (E-H) Behavioral analysis of lines in three gene categories captured in the pigmentation screen, including locomotion (E), sleep latency (F), sleep bout duration (G), and total sleep (H). Individual t-tests were performed to assess if genes in a category were significantly different to all other genes tested, and only those with a $p<0.10$ are reported, with *=$p<0.05$.

Taken together, 77 of our 132 conserved genes are likely to have a neurological function, because they are implicated human neurological disease (52 conserved genes) or were classified as a behavioral "outliers" (45 conserved genes).

### 11 genes alter dopamine levels in the fly head

To prioritize our gene list for dopamine measurement, we identified conserved genes (132 conserved/152 pigmentation genes). Then, we prioritized these based on whether knockdown of these genes showed a strong pigmentation effect (38 genes), a behavioral effect in the DAM (45 genes), and/or a human neurological disease association (52 genes). 72% (95/132 conserved genes) were represented in one of these categories, and 29% (39/132) were represented in at least two (Fig 4A). We classified genes in two or more categories as priority hits and tested them for changes in dopamine using High Performance Liquid Chromatography (HPLC). We repeated previous results showing that knocking down *TH* or overexpressing a *TH* cDNA using *TH-GAL4* significantly reduces or increases head dopamine without effecting serotonin (Figs 4B and 4C) (29).

## Figure 4

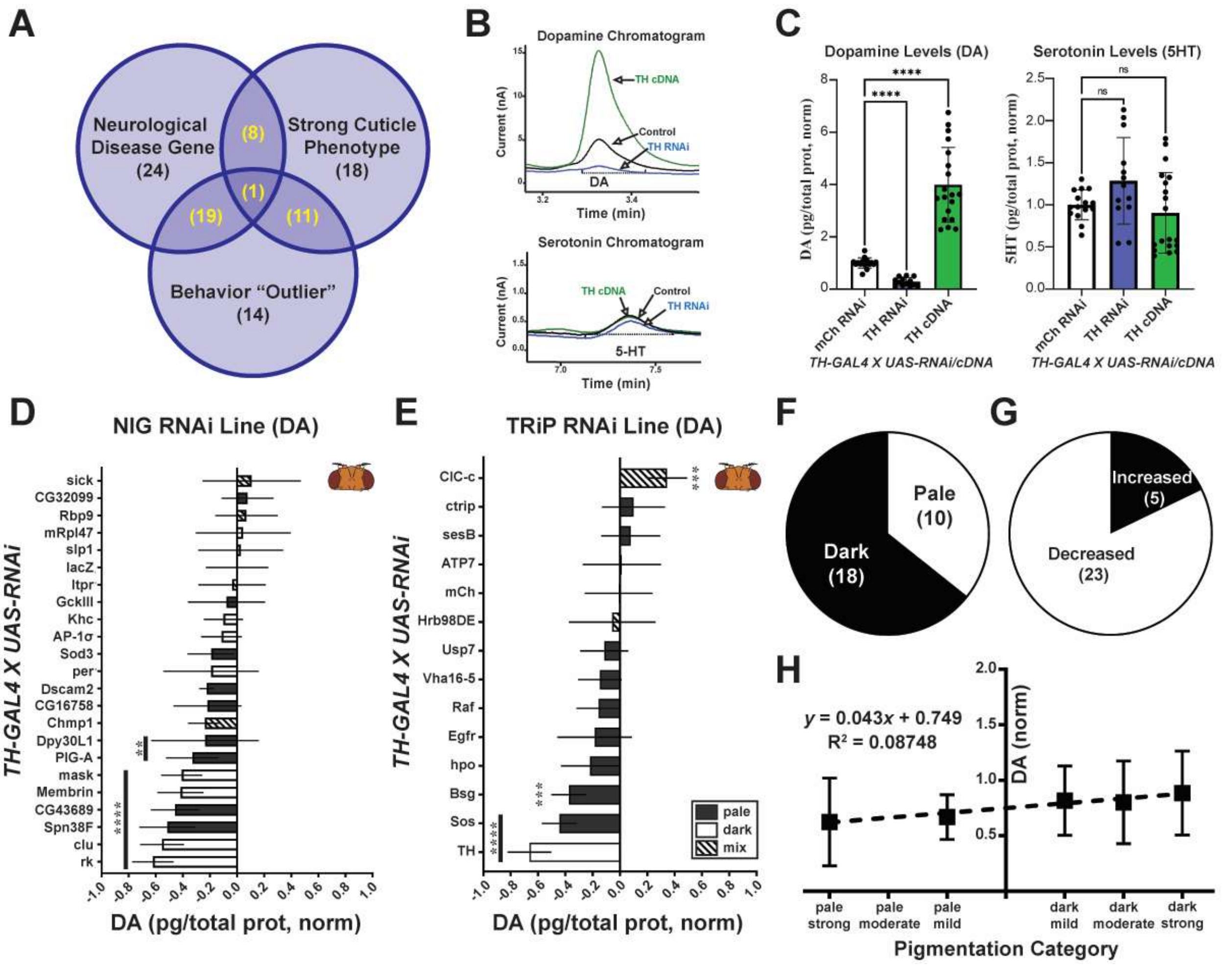

**Fig 4. Eleven genes alter head dopamine upon knockdown.** (A) Venn diagram of the categories used to prioritize 39 genes (in yellow). (B) Representative chromatograms for dopamine (DA) and serotonin (5-HT) upon knockdown or overexpression of TH in Dopaminergic cells (*TH-GAL4*). (C) Quantification of DA and 5-HT from chromatograms. (D-E) HPLC analysis on prioritized genes for UAS-RNAi lines from NIG (D) and TRiP (E). Pie charts for all genes for cuticle color (F) and trend in dopamine (G). (H) XY plot comparing pigmentation phenotype to dopamine levels. For (C) Brown-Forsythe ANOVA test performed with Dunnett's T3 multiple comparisons test. For (D) and (E) ordinary One-way ANOVA test with Dunnett's multiple comparisons test. **=p<0.01, ***=p<0.001, ****=p<0.0001. Error bars represent SD.

We found that 11 of 35 prioritized genes (note: some not tested due to lethality or stock issues) significantly altered head dopamine levels (Figs 4D and 4E). Since control NIG lines showed about ~80% dopamine levels compared to the TRiP collection (S3 Fig), we compared the DA levels to control lines appropriate for each collection (e.g. UAS-lacZ RNAi). Unlike cuticle pigmentation, where 65% of genes caused a dark cuticle (excludes mixed cuticle phenotype), most genes trended toward a reduction in dopamine (71%, 25/35, Figs 4F and 4G). There was no correlation between the cuticle color and the dopamine level in the fly head (Fig 4H).

In summary, our HPLC analysis on fly heads revealed 11 genes that significantly affect dopamine levels. We saw a trend in reduction in dopamine across all lines tested, and we observed no correlation between cuticle color and dopamine level.

### Brain study reveals *mask* and *clueless* as potential regulators of brain dopamine

To determine which of the 11 genes that affect dopamine in the head have consequences on the brain, we examined the expression of four of them in the brain using available T2A-GAL4 lines (39–41). We found that several overlapped with somewhere between 25% - 100% of DANs (S5 Fig, Figs 5B and 5G). In publicly available single-cell mRNA sequencing datasets, ten of our gene hits (all except *Spn38F*) show expression in the brain with varying overlap with TH expressing DANs (42,43).

## Figure 5

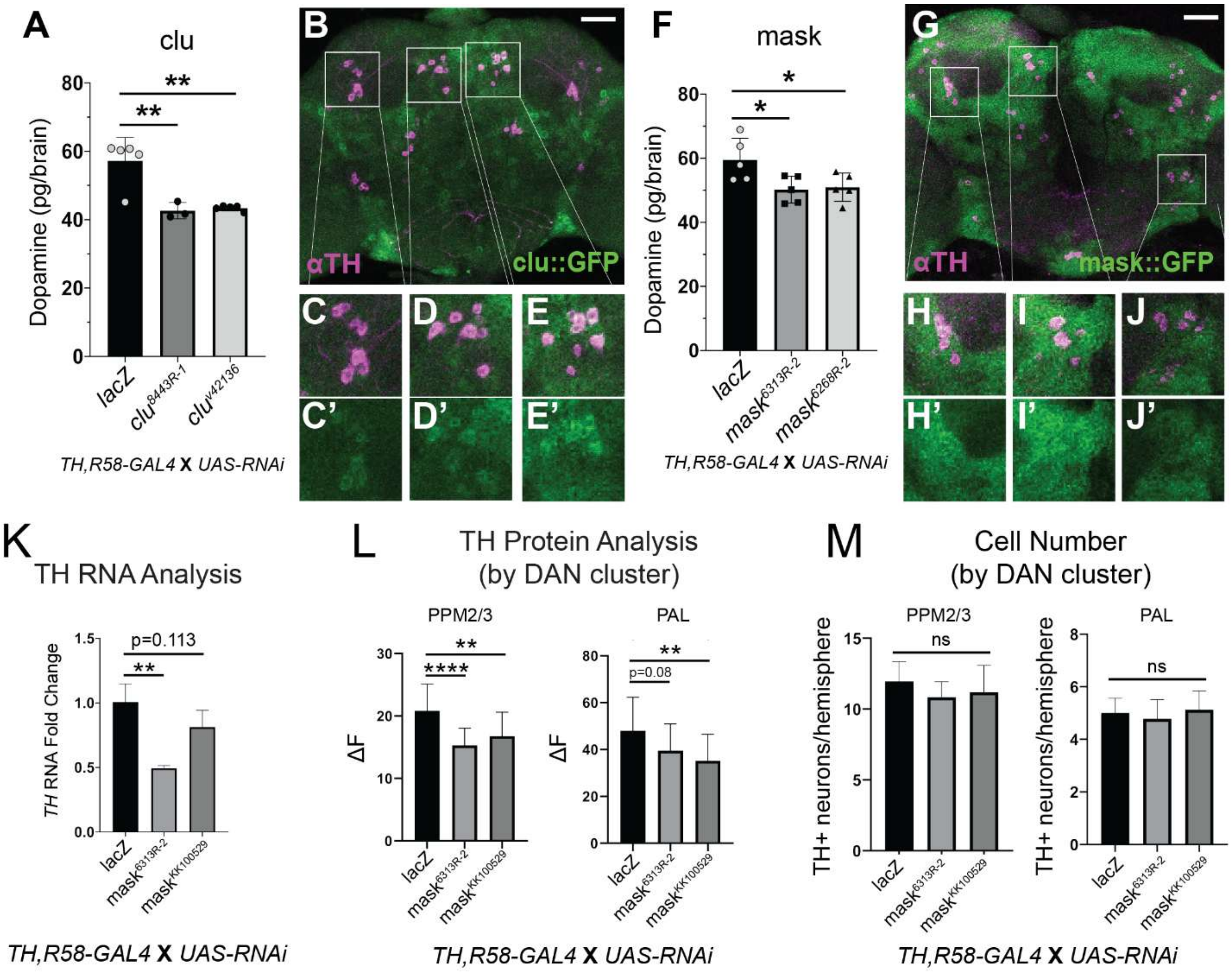

**Fig 5. Brain dopamine analysis identifies *mask* and *clu* as novel regulators of dopamine levels in the fly brain** (A) Total dopamine in the adult fly brain upon knockdown of *clu*. (B) *clu::GFP* expression pattern in the adult brain (B-E, B'-E'). (F) Total dopamine in the adult fly brain upon knockdown of *mask*. (G) *mask::GFP* expression pattern in the adult brain (H-J, H'-J'). (K) qRT-PCR of TH upon *mask* knockdown with the recombined DAN (*TH, R58-GAL4*) driver. (L) Quantification of total TH protein level through immunofluorescence in DAN clusters upon knockdown of *mask*. (M) Quantification of TH positive (TH+) neurons in DAN clusters upon knockdown of *mask*. Ordinary one-way ANOVA was done with Dunnett's multiple comparisons test (F, I, N). Error bars represent SD. Scale bars are 50 μm.

The *TH-GAL4* line commonly used for dopamine studies misses a large portion of DANs, particularly in the PAM cluster (~87 DANs missed/~142 total DANs per hemisphere) (44), making it not ideal for quantifying total changes in brain dopamine. We recombined the *TH-GAL4* with another driver that hits a large portion of the PAM cluster neurons (*GMR58E02-GAL4 or R58-GAL4)* (18) generating a recombined line (labelled *TH,R58-GAL4*) that hits ~95% of DANs (S4 Fig).

We microdissected adult fly brains and measured dopamine for our 11 gene hits. Most of them showed no change in brain dopamine (S5 Fig), but *mask* (*multiple ankyrin repeats single KH domain*) and *clueless* (*clueless*) knockdown reduced total brain dopamine for two independent RNAi lines (Figs 5A and 5F). Protein trap lines (45,46) for both Clueless and Mask show broad expression patterns in the brain (Figs 5B-5EJ-5M and 5G-5J). When we focused on DAN clusters (e.g. PPL1 and PPM2), Clueless protein appears to be enriched in DANs (Figs 5C-5E and 5C'-5E'), whereas Mask shows a broad and equal expression throughout most cells in the brain (Figs 5H-5J and 5H'-5J').

In summary, our fly brain analysis revealed two genes that (18%, 2/11) significantly altered brain dopamine.

### ***mask*** **regulates Tyrosine Hydroxylase and alters sleep in a dopamine-dependent manner.**

To test if *clu* and *mask* knockdown could be due to DAN loss, we quantified the number of DANs in several clusters (44). There was no difference in neuron number for every cluster examined (Fig 5M and S6 Fig). Since our pigmentation gene study revealed that only the manipulation in TH reduced brain dopamine (Fig 1), we examined if knocking down *clu* or *mask* affects *TH* RNA or protein level. Knockdown of *mask* led to a significant reduction in *TH* mRNA

level for one RNAi (50% reduction), with the other RNAi trending (20-30% reduction, Fig 5K). Upon examining TH levels in different DAN clusters, two clusters showed significant reduction in TH protein level, with other clusters showing a trend in TH protein reduction (PAL and PPM2/3, Fig 5L and S6 Fig). To assess behavioral consequences, we performed additional sleep analysis on *mask*. Dopamine is a wake-promoting agent, and complete loss of dopamine in the brain significantly increases sleep (47,48). Knocking down *TH* using the *TH-GAL4* did not significantly alter total levels of sleep during light or dark periods (S7 Fig), which may be due to the selective expression of the GAL4 or the partial reduction of dopamine (Fig 4C). However, there was a significant increase in sleep in the two hours before light onset, which is classified as light anticipation. Similarly, knockdown of *mask* with the TH-GAL4 showed a highly consistent reduction in light anticipation (Fig 6A, 6B, 6B', and S8 Fig). When we feed the flies L-DOPA, the effect is no longer seen (Fig 6C, S8 Fig). Additionally, in *Drosophila* caffeine's effects on sleep are mediated by dopamine through a point that is upstream of L-DOPA, potentially via TH activity (49). Based on this, we suspected that *mask* knockdown would ameliorate the effects of caffeine on sleep, since *mask* appears to reduce the synthesis of *TH*. We saw that caffeine's effect on total sleep and sleep in the dark is ameliorated upon *mask* knockdown (Fig 6D, 6E, and S8 Fig).

## Figure 6

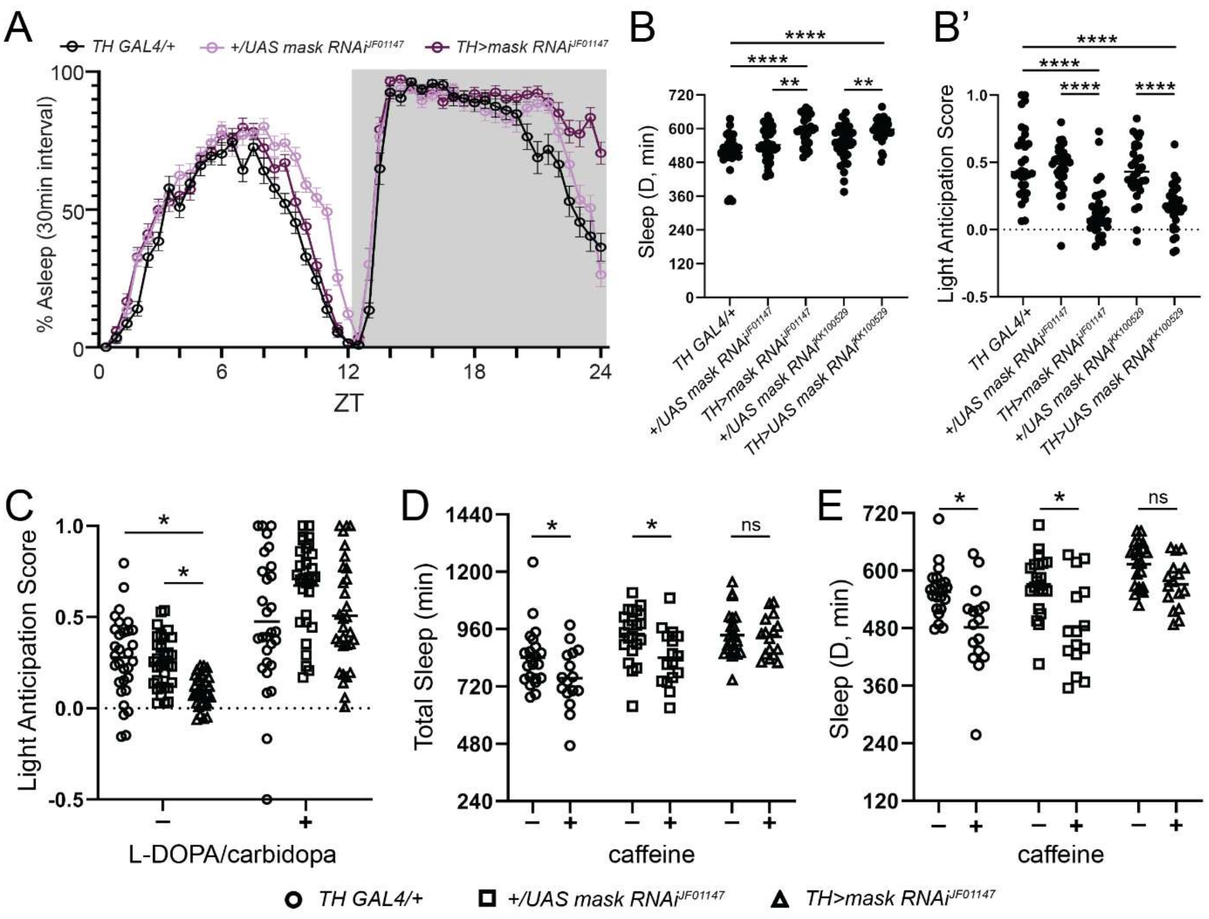

**Fig 6. *mask* knockdown reduces TH mRNA and protein and causes sleep phenotypes that align with reduction in TH levels** (A) 24-hr sleep graph (sleep binned in 30-minute intervals) upon mask knockdown using the TH-GAL4. (B) Quantification of total sleep throughout the 12-hr dark period upon mask knockdown (B') Quantification of light anticipation upon mask knockdown. (C) Light anticipation in mask knockdown flies with L-DOPA feeding. (D-E) Caffeine effects on sleep upon mask knockdown – (D) Total Sleep and (E) Sleep during the 12-hr dark period. For samples with more than one experimental, ordinary one-way ANOVAs were performed with Dunnett's multiple comparisons. (B, B', C). For samples with one experimental, individual t-tests were performed (D,E). *=p<0.05, **p<0.01, ****p<0.0001. Error bars represent SD.

Follow up studies on *clueless* showed that *clueless* knockdown increases TH RNA level by about 1.5-2X, with no effect on TH protein level or TH+ neurons (S9 Fig), and while there was an increase in sleep across several RNAi lines, there is no light anticipation phenotype. Feeding TH>clu RNAi flies L-DOPA was unable to rescue the *clueless* effects on sleep (S9 Fig), suggesting that the effects on dopamine and TH may be a secondary effect. Additionally, the antioxidant NACA was also unable to rescue the sleep defects seen upon *clueless* knockdown.

In conclusion, *mask* appears to reduce total dopamine levels and sleep by affecting transcription of TH, while *clueless* is acting through an alternative mechanism.

## Discussion

### Validation of 148 novel cuticle pigmentation genes

Our RNAi screened confirmed 153 genes that were previously implicated in *Drosophila* pigmentation – 148 of these genes have not been previously associated with dopamine biology. Because we observed a strong enrichment for genes involved in pigmentation and tyrosine metabolism (S1 Fig), our method for screening and analysis effectively identified genes that play critical roles in the biological process of interest.

While prior research has compared genes that overlap with different types of screening strategies (e.g. chemical mutagenesis vs. RNAi), most RNAi phenotypes from large-scale screens have not been validated with alternative RNAi lines or collections. The original source RNAi screen used the lines available at the VDRC stock center (35,37). Alternative sources for RNAi lines (i.e. TRiP and NIG) start with different backgrounds, vectors, hairpin lengths, insertion methods, and/or target different areas of the gene (36,50). This allow researchers to overcome the off-targeting and weak silencing seen with RNAi analysis, but to our knowledge, there have been no screens or meta-analyses to systematically test consistency across lines from different sources. In this study, we found that ~46% (153/330) of genes with a pigmentation phenotype showed a phenotype in an alternative RNAi, and 87% of those phenotypes showed agreement in pigmentation color, highlighting that pigmentation as a phenotype is relatively consistent (S10 Fig).

Interestingly, 85% of our pigmentation genes had at least one homolog, whereas genes tested in the original RNAi screen list only had ~68% conservation (1.25-fold enrichment, S2 Fig). Even though pigmentation of the cuticle through dopamine and its synthesis enzymes, produced through TH and Ddc, is an invertebrate-specific phenomenon (12), the enrichment for conserved genes suggests that this phenotype is relevant for some conserved biological pathway and mammalian biology. We propose that cuticle pigmentation in insects may be a “phenolog” (51) of certain neurological traits in mammals

### Protein-protein interaction network highlights unexpected gene associations

When we investigated what kind of gene categories were found in the pigmentation screen, we saw clusters of genes that are associated with ubiquitin-proteasome system (UPS), RNA processing, mitochondria, and developmental signaling pathways. Each of these processes could be relevant to dopamine biology. For example, there is some evidence that the UPS system regulates TH levels in the mammals since inhibition of the UPS system has been shown to increase the TH protein in a PC12 rat cell line (52,53). In addition, changes in RNA processing could indicate changes in the dopamine synthesis enzymes TH and Ddc since these enzymes undergo RNA processing to create two different isoforms (54). The two TH isoforms differ in tissue expression and kinetics(55). In mammals, post-transcriptional trafficking of TH mRNA and translational regulation has been suggested to be important for its function (56).

We identified 12 genes that are known to function at the mitochondria, mostly in the electron transport chain (*ND-13B*, *ND-42*, *COX5A*), mitochondrial mRNA translation (*mRpL47*, *mRpL48*, *CG4679*), and mitochondrial protein localization (*CG8728*, *clu*, *mask*, *Mpcp2*). It is well-known that the mitochondria have a key role in dopamine metabolism in mammals since MAO acts in the mitochondria to degrade dopamine. However, there is no known MOA homolog in flies(12). Interestingly, the metabolites for oxidation and methylation of dopamine (DOPAC and HVA) have been observed in flies (57–60). In addition, mutants for enzymes thought to be the primary regulators of dopamine degradation (*ebony*, *tan*, and *speck*) (61,62) did not have any effect on dopamine in the brain (Fig 1), suggesting an alternative process regulates dopamine degradation in the brain. It is plausible that mitochondria could be involved in this process.

The last major group of genes identified from our screen was developmental signaling, specifically EGF signaling and Hippo signaling. To date, neither EGF nor Hippo signaling have an established connection with dopamine biology in *Drosophila*. We may have pulled these genes out is because the process of forming the dorso-central thorax is developmentally regulated (63–65). However, we did not identify core components of the other key pathways, such as Notch or Hedgehog, suggesting that EGF and Hippo are more specifically involved in this pigmentation process.

### Cuticle pigmentation phenotypes may not reflect changes in dopamine

The field has generally assumed that a darker cuticle reflects an elevated level of dopamine (15,66). However, when we measured dopamine from degradation enzyme mutants using HPLC, we saw either no effect on dopamine level or reduced level of dopamine in the head. In addition, when we measured dopamine from the pigmentation genes from the screen, a darker cuticle did not reflect elevated levels of dopamine in the head. While we were able to increase dopamine by 3-5 fold upon TH cDNA overexpression in the head (Fig 4C), this phenomenon was not observable in the brain. One reason could be that there is a selective pressure or a strong feedback mechanism that prevents elevated levels of dopamine in the nervous system. Since oxidized dopamine could be toxic (67), this could be a mechanism to prevent cell toxicity. Regardless, changes in cuticle pigmentation do not necessarily reflect changes in dopamine level, so assumptions should not be made that increase in melanin levels in the fly corresponds to increased levels of dopamine.

Upon seeing alterations of dopamine in the head for some of the classic pigmentation genes, we next wanted to test if this corresponded to changes in the brain. In the case of established dopamine metabolism genes, we only saw a change in dopamine levels upon reduction of TH. When we looked at expression for some of these genes, we saw that all the genes we were able to assess (*black*, *speck*, *tan*) were expressed in the brain, but their expression did not overlap well with DANs. It may be that dopamine degradation does not happen in the neurons since it was hypothesized to occur in the glia (31,68). However, the expression does not seem to show a broad glial expression using the reagents we had access to. For the 11 genes from the pigmentation RNAi screen that showed altered levels of dopamine, only 2 (18%) of them showed an effect on dopamine in the brain. Hence, head dopamine levels should not be used as a proxy for brain dopamine levels.

### *clu* and *mask* reduces brain dopamine levels through different mechanisms

Once we identified that knockdown of *clueless* and *mask* both reduced dopamine in the brain without affecting the number of DANs, we hypothesized that they may be acting through TH since our initial dopamine metabolism studies showed that only changes in TH significantly affected dopamine levels in the brain.

*mask* encodes a scaffolding molecule that functions in multiple contexts (69–71). In this study, *mask* knockdown reduced both *TH* mRNA and protein levels, suggesting it acts on TH transcription. Follow-up behavior analysis, showed that *mask* knockdown in DANs led to a highly consistent change in sleep. Specifically, *mask* knockdown reduced the number of flies that anticipated light. While fruit flies typically start to wake before light onset (72), loss of mask in DANs suppresses this anticipation behavior. Supporting this mechanism, we saw a similar phenotype with the TH RNAi and the DAN driver (TH>TH RNAi, S7 Fig). Dopamine is a wake-promoting molecule, and if dopamine synthesis normally increases before light onset as a means of waking the fly, reduced levels of TH could put strain on this mechanism.

When we fed *mask* knockdown flies the precursor for dopamine, L-DOPA, this abolished the effect on light anticipation. We suspect that feeding L-DOPA does not override all sleep-dependent effects because clueless sleep phenotypes remained when we fed them L-DOPA (S9 Fig). There is evidence that in the fruit fly caffeine affects sleep through TH. Specifically, Nall et al. showed that caffeine's effect on sleep is absent in TH mutant flies, even if L-DOPA is given (49). Since *mask* knockdown effects TH mRNA levels, we tested if mask knockdown would suppress the effects of caffeine. We found that the reduction in total sleep and sleep during the dark period normally seen upon caffeine administration were absent upon mask knockdown. This further supports that mask alters TH levels.

While we do not have any direct evidence for how *mask* may be altering TH levels, *mask* has been implicated in multiple biological pathways that were highlighted from our screen, including EGF signaling (e.g. *Egfr*, *Sev*, Raf), Hippo signaling (e.g. *hpo*, *wts*, *mats*), and mitochondrial biology (73–76). Egf and Hippo signaling have well-known transcriptional effects (77,78), but whether they may regulate TH transcription directly or indirectly in this context need further investigation. It is also possible that *mask* is acting in several of these pathways at once, which causes it to have a strong additive effect that produces a significant change in dopamine in the whole brain, while knockdown of individual pathway genes may have a more restricted role (e.g. *Egfr*, *Sos*, *hpo*).

In addition to *mask*, we also saw that knockdown of *clueless* leads to significant reduction in dopamine. However, when we explored the behavioral consequence of *clueless* knockdown, it did not show the same effect on sleep as *mask* or *TH* knockdown. In addition, the sleep phenotype was not rescued by L-DOPA, and molecular biology showed that *clueless* knockdown leads to an increase in TH mRNA with no change in TH protein. Taken together, this indicates that the effect on dopamine from *clueless* knockdown may be a secondary consequence. *clueless* encodes a ribonucleoprotein thought to act at the outer mitochondrial membrane to promote proper formation of protein complexes including mitophagy proteins like Pink1 and Parkin (79,80). It also interacts with the ribosome and translocase complex at the mitochondrial membrane (79,81) suggesting it acts as a regulator for mitochondrial protein translation and import. Knockdown of *clueless* showed no effect on TH protein level, but since this is a mitochondrial protein, it may be more likely that the effect on dopamine is through the previously proposed roles of mitochondria in regulating dopamine levels.

In conclusion, while both genes cause a reduction in dopamine, *mask* appears to regulate dopamine by altering TH levels, possibly through regulation of TH mRNA. On the other hand, *clueless* effects on dopamine appear to be a secondary consequence, with a behavioral effect that is independent of dopamine. Additional analysis on changes in enzymatic activity as well as potential downstream targets would help identify how these genes are acting.

### Some regulators of dopamine levels may act locally

When we examined the expression of some of our pigmentation genes in the brain (S11 Fig), we found that many of them do not overlap with all DANs. Indication that measuring global changes in dopamine may not be best for determining effects on DA for many of these genes. They may require targeted dopamine measurements, perhaps through a fixed or live reporter (82,83). It is also possible to assess changes in dopamine using behavior, but this would require detailed assessment for each gene since behaviors are often cluster specific (84).

In conclusion, our screen of identified novel pigmentation genes, a subset of which were identified as novel regulators of dopamine *in vivo*. Unbiased forward genetic screens in model organisms are powerful ways to identify unanticipated links between distinct biological pathways and provide new molecular handles to study the functional connections between them. Application of such strategies to the regulation of dopamine levels will likely continue to identify novel factors, some of which will impact our understanding of human neurological and neurodevelopmental diseases.

## Resource Availability

All data generated or analyzed during this study are included in this published article and its supplementary information files. This study did not generate any new unique reagents.

## Acknowledgements

We thank the Bloomington *Drosophila* Stock Center (USA), the National Institutes Genetics Fly Stock Center (Japan), *Vienna* Drosophila RNAi Center (Austria) and Dr. Hugo Bellen for providing useful fly stocks and reagents for this project. We would like to thank Drs. Jonathan Andrews,

Hugo Bellen, Brigitte Dauwalder, Herman Dierick, Linsday Goodman, Oguz Kanca, Kartik Venkatachalam, Michael Wangler, Sheng Zhang and Huda Zoghbi for useful suggestions, discussions and advice on the overall project. This work was supported by startup funds to SY from the Jan and Dan Duncan Neurological Research Institute at Texas Children's Hospital and the Department of Molecular and Human Genetics at Baylor College of Medicine. Confocal microscopy at BCM was supported in part by the Intellectual and Developmental Disabilities Research Center (IDDRC, grant number U54HD083092) from the Eunice Kennedy Shriver National Institute of Child Health & Human Development.

## Authors Contributions

S.Y. and S.L.D. conceived the experiments and wrote the manuscript. S.Y., E.S.S., D.B., and S.L.D. conducted the pigmentation screen and DAM behavior analysis screen. S.L.D. and K.W. conducted the pigmentation mutant analysis experiments. S.L.D., S.B.G, H.D-S, Y.F., and K.W. conduced the HPLC analysis.  S.L.D. and Y.F. performed confocal imaging. S.L.D. conducted all data analysis.  All authors participated in the critical analysis of the manuscript.

## Declarations of Interests

The authors declare no competing interests.

## Materials and Methods

### Fly Maintenance

Flies (*Drosophila melanogaster*) strains and crosses were reared at 25°C on a 12:12 Light/Dark (LD) program unless stated otherwise. Flies were fed a molasses-based food source. Different temperatures were used to achieve different levels of gene knockdown or overexpression, which is documented for each experiment. Flies were transferred to a new vial 0-2 days post-eclosion (dpe) for age-appropriate analysis. Experiments were run on 3-7 dpe flies unless otherwise stated.

## Fly Stocks and Crosses

Mutant and transgenic trains were obtained from Bloomington *Drosophila* Stock Center (BDSC, https://bdsc.indiana.edu/), Vienna Drosophila Research Center (VDRC, https://www.viennabiocenter.org/vbcf/vienna-drosophila-resource-center/), the Japanese National Institute of Genetics (https://shigen.nig.ac.jp/fly/nigfly/), or were gifts from scientists in the field or generated in house (S1 Table – S5 Table).

For the cuticle screen *pnr-GAL4* (85) was crossed to the specified UAS-RNAi line (see S1 Table) at 29°C, 25°C, and/or 18°C and examined after pigmentation is completed (>1 dpe). For the behavior and HPLC analysis *TH-GAL4* females (also called *ple-GAL4*, BDSC stock #8848) were crossed to the specified RNAi line (see S2 Table). For all HPLC analysis, the controls were specific to the tested UAS-RNAi line (86). Thus, the TRiP collection of RNAi lines are compared to the *UAS-mCherry(mCh) RNAi* line (BDSC #35785) and the NIG collection of RNAi lines as well as the VDRC RNAi lines are compared to the *UAS-lacZ RNAi* line. For the HPLC analysis performed on brains, we recombined the *TH-GAL4* with another DAN driver *GMR58E02-GAL4* (BDSC #41347), which expresses in a large group DANs in the PAM cluster (27). These flies are referred to as *TH,R58-GAL4* throughout the article. The *TH,R58-GAL4* line was also used for qPCR and TH protein analysis via immunohistochemistry. For gene expression analysis *UAS-mCh::nls* (BDSC #38424) females were collected and crossed with respective T2A-GAL4 lines (see S5 Table). Female and males were both imaged at ~5-7dpe. For the follow up sleep studies, the *UAS-RNAi* males were crossed with *TH-GAL4* virgins for the experimental. For the controls $w^{1118}$ females were crossed to males from either the *TH-GAL4* and *UAS-RNAi* lines independently.

### Notum Dissection and Imaging

For thorax analysis and dissection, males and females were both selected, though differences were not generally observed, and females are shown here. Flies that were 3-5 dpe were collected and placed in 70% EtOH. Notum dissection and imaging was previously published (87). In summary, the thorax was dissected by removing the legs, abdomen, and head. Then the thorax was cut such that a hole was on the ventral side to allow solution to pass into the thorax. These dissected thoraces were placed in 10% KOH for 10 minutes at 90°C on a heat block. Then, the KOH was removed and replaced with 70% EtOH solution. They were mounted on a slide prepared with tape on two sides in mounting media (50% glycerol, 50% EtOH). The thoraces were imaged on a stereo microscope (Leica MZ16) using OPTRONICS® MicroFIRE camera. The images are z-stack brightfield images taken and collapsed using extended depth of field in Image-Pro Plus 7.0 and In-Focus (Version 1.6).

### Brain Expression Analysis

Brain dissections and imaging were performed as previously reported (88). In summary, the brains were dissected at 3-7 dpe in ice cold PBS and then fixed in 4% PFA in 0.5% PBST for 20 minutes. Then, they were washed with a quick wash in 0.5% PBST followed by three 10-20-minute washes in 0.5% PBST while on a rotator. The samples were placed in primary antibody solution [anti-Tyrosine Hydroxylase 1:500, PelFreez Biologicals, rabbit, P40101; anti-elav 1:100, Developmental Studies Hybridoma Bank, rat, 7E8A10 in solution (5% Normal Donkey Serum, 0.1% $NaN_3$ in 0.5% PBST)] at 4°C for 3 days. Then, the samples were washed with a quick wash in 0.5% PBST followed by three 10-20-minute washes in 0.5% PBST while on a rotator. After the last wash, the samples were placed in a secondary antibody solution [anti-Rabbit 1:200 (Thermo Fisher Sci., Alexa-647, A-21208); anti-rat 1:200 (Thermo Fisher Sci., Alexa-488, A-27040) in solution (5% Normal Donkey Serum, 0.1% $NaN_3$ in 0.5% PBST)] for two hours at room temperature. The samples were washed again with one quick wash followed by three 10-20 minutes washes in 0.5% PBST on a rotator. Then, the brains were mounted in Vectashield mounting media and imaged on a confocal microscope (Zeiss LSM 710 or Zeiss LSM 880). All images shown in this manuscript are Z-projection images that were generated using the ZEN software (Zeiss).

### RNAi-based Pigmentation Screen

We identified pigmentation gene hits from a primary screen performed in Jurgen Knoblich's lab using the Vienna Drosophila Resource Center collection of RNAi lines by accessing their public RNAi screen database (https://bristlescreen.imba.oeaw.ac.at/start.php). The original screen was performed on 20,262 RNAi lines, encompassing 11,619 genes (~82% of protein-coding genes) (22). We selected genes that showed a cuticle pigmentation score for any RNAi line tested (gene scores ranged from two to ten). To validate pigmentation defects, we selected genes that had RNAi lines within the Fly National Institute of Genetics (NIG, 220 genes, 426 RNAi lines) or the Harvard Transgenic RNAi Project (TRiP, 221 genes, 292 RNAi lines) collections. Each RNAi line was tested at 29°C and 25°C. If there was no phenotype no further testing was performed. If it was lethal, we tested it at 18°C.

### Classification of Cuticle Phenotypes

Phenotypes were scored using a qualitative scoring system. This system used *UAS-TH RNAi* and *UAS-ebony RNAi*, some of the strongest regulators of cuticle color, as a baseline for the "strong". Then, the other lines were placed along the spectrum as mild, moderate, or strong. Phenotypes were scored by two independent observers and differences were settled by a third independent observer. If there was variability with one line or if there was variability amongst lines, this might appear as mild-moderate, moderate-strong, or mild-strong. In these cases, the strongest phenotype observed was documented as the recorded phenotype for a given gene.

### Protein-protein interaction network and gene ontology analysis

The protein-protein interaction network was generated using STRING (search tool for recurring instances of neighboring genes; https://string-db.org/). This database includes protein-protein interactions that are physical interactions and functional associations. For our data set we included these sources of interactions: gene neighborhoods (genes that are found close together across species) (89), curated databases (publicly available databases of protein interactions), and experimental evidence (known complexes and pathways from curated sources) (90,91). Any genes that did not interact with other genes from our screen were not included in the interaction network. This database was last accessed on 05/05/2023. The lines between genes represents the confidence in the interactions, which is based on complex algorithms that are dependent on the source.

The gene ontology analysis was generated using GOrilla (Gene Ontology enRIchment anaLysis and visuaLizAtion tool, https://cbl-gorilla.cs.technion.ac.il/). The 153 pigmentation genes hits were included as target genes, and the background genes were the entire VDRC RNAi collection included in the Mummery-Widmer et al. 2009 screen. Only the GO terms with a p-value greater than 0.001 and a fold enrichment of greater than three were included in the analysis. Redundant terms were removed using Revigo (http://revigo.irb.hr/) with a stringency of 0.7. Those that have a fold enrichment greater than five were included in S1 Figure.

### Human Neurological Disease Gene Classification

Homologs for each fly gene were identified using DIOPT (DRSC Integrative Ortholog Prediction Tool, v8.0, https://www.flyrnai.org/diopt) (92). Genes were classified as orthologs if they had a DIOPT score≥3. The human genes were scored as neurological disease-causing genes if they had an OMIM (Online Mendelian Inheritance of Man, last accessed 01/01/2023, https://www.omim.org/) disease association with any documented neurological phenotype (93). Genes were also classified as human neurological disease genes if they were in the Simons Simplex Collection of Autism Spectrum Disorder gene list (last accessed 06/01/2022, https://gene.sfari.org/) and had a score equal to or less than 3 (1, 2, or 3) and/or syndromic (S) (94).

## Behavior Analysis using the *Drosophila* Activity Monitor

### Behavior screen

For the behavior screen, the crosses were set in a 12:12 LD chamber at 25°C and transferred every 2-3 days to increase the number of progenies. Upon eclosion, flies were transferred to 29°C and kept for 2-3 more days before testing. Individual tubes (PPT5x65 Polycarbonate, Trikinetics Inc, USA) appropriate for use with the *Drosophila* Activity Monitor (DAM, Model DAM2 for 5mm tubes, TriKinetics Inc, USA) were loaded with approximately ½" worth of molasses-based food. The end of the tube with food was sealed by placing the vial into Paraplast® (Sigma-Aldrich) wax three times and allowing it to dry. Once they were dry, individual male flies were placed into each vial, the vial was placed into the DAM recording chamber, and it was sealed using a 5mm tube cap (CAP5, TriKinetics Inc, USA) with a hole stuffed with cotton. For each genotype, 16 individual males were run, except in a few cases where we were unable to get that many flies. In which case, as many living males as possible were run. The flies were loaded into a 12:12 LD chamber at 29°C and monitored for 3 days. Then, the flies were removed and dead flies were eliminated from analysis.

Locomotion and sleep analysis was run on a 24-hour period, which started at the first onset of light after the flies were placed in the chamber (i.e. 18-24 hours after being placed in the chamber). Locomotion was calculated based on the number of beam crossings over the full 24 hours, the 12 hours of light, and the 12 hours of dark. Sleep was classified as 5 minutes without any beam crossings. Total Sleep was quantified as total time spent sleeping over the 12 hours of light and the 12 hours of dark. Sleep latency was quantified as the amount of time after light onset or dark onset before a 5-minute period of sleep. Sleep Bout Length was quantified as the average length of sleep bouts (>5-minute period of sleep) during the 12 hours of light and 12 hours of dark.

“Outliers” were classified as lines that were more than two standard deviations from the mean of all lines for any of the phenotypes scored (24-hour locomotion, 12-hour light locomotion, 12-hour dark locomotion, 12-hour light total sleep, 12-hour dark total sleep, 12-hour light sleep bout length, 12-hour dark sleep bout length, sleep latency during the light, or sleep latency during the dark).

### Mask follow up behavior studies

For these studies, similar tools and settings were used with these core exceptions. Upon eclosion flies were transferred and kept at 25°C in group housing. For the behavior without drugs, individual males were placed in tubes with sucrose food (2% agar + 5% sucrose) at 3-6 dpe and behavioral analysis was collected and analyzed for the following five days. Sleep was then averaged across the five days for each individual fly before statistical analysis was performed. For the behavior with drugs, similar conditions were followed, except data was collected on the 2-6 days after they were loaded into behavior tubes to allow the drug to take effect. The following drug doses were used for their respective experiments (L-DOPA: 3mg/mL L-DOPA + 12.5 µg/mL carbidopa; caffeine: 0.5 mg/mL caffeine; NACA: 40 µg/mL N-acetylcysteine amide antioxidant). Drugs were dissolved directly into the sucrose food except for carbidopa which was dissolve in $H_2O$ at 1:50x concentration and then added into the sucrose food.

## High Performance Liquid Chromatography (HPLC) analysis

### Sample Preparation

For the HPLC on fly heads and brain, we adapted and modified the protocol previously published (95,96). Crosses were reared at 29°C and flies were transferred into new tubes 0-2 days post eclosion (dpe). They remained at 29°C until they were 3-5 dpe. Female heads were collected by anesthetizing flies with $CO_2$ and cutting their heads off with a razor blade. Samples were collected between ZT04-ZT08. The heads were placed into 60 µL of 50 mM citrate acetate (pH=4.5) and either used for analysis that day or frozen at -20°C. Five heads were used per sample, and ~10 samples were run per genotype. The day of HPLC analysis, the samples were thawed and grounded using a pestle for 30 seconds (Cordless Pestle Motor and Fisherbrand™ Disposable Pellet Pestle for 1.5mL tube). Then the samples were spun down at 13,000 rpm for 10 minutes. The supernatant was removed and placed into a new vial. 10 µL from each sample was used for the Bradford Protein Analysis Assay. The rest of the sample solution (~40-50 µL) was loaded into a vial (300 µL Polypropylene Sample Vials with 8mm Snap Caps) for HPLC analysis.

For HPLC analysis on fly brains, flies were collected 3-7 dpe. Then, 10 fly brains (5 male, 5 female) were dissected in ice cold PBS. Right after dissection, the brains were transferred to 60uL of ice cold 50mM citrate acetate (pH=4.5). Samples were frozen at -20°C and ran within 4 weeks of collection. The day of HPLC analysis, the samples were homogenized similarly to the fly heads, though for the Protein Assay, 20 µL of sample was used. Then, the remaining solution was loaded for HPLC analysis.

### Bradford Protein Analysis Assay

The Bio-Rad Bradford Assay was used for colorimetric scoring of total protein. See product details for full description (Bio-Rad Protein Assay Kit I #5000001). Briefly, the dye reagent was diluted 1:4 in MilliQ $H_2O$. Then, the solution was filtered through a 0.22 µm SFCA Nalgene filter. 10 µL of each protein sample (standard or fly sample) was placed into single wells of a 96-well plate. Then, 200 µL of diluted 1:4 dye reagent was added to each well. The samples rested for 30-45 minutes and then absorbance was measured using BMG Labtech FLUOstar OPTIMA microplate reader. Protein measurements for each sample were calculated based on standards ran on the same plate.

### HPLC Information

The HPLC used is an Antec® Scientific product with a LC110S pump, SYSTEC OEM MINI Vacuum Degasser, AS110 autosampler, a SenCell flow cell with salt bridge reference electrode in the Decade Lite. Data was collected and processed using DataApex Clarity™ chromatography software. The column used is chosen to work well for neurotransmitters (Acquity UPLC BEH C18 Column, 130Å, 1.7 µm, 1 mm X 100 mm with Acquity In-Line 0.2 µm Filter). The mobile phase was a 6% Acetonitrile mobile phase optimized for our samples (74.4 mg $NA_2EDTA \cdot 2H_20$, 13.72 mL 85% w/v phosphoric acid, 42.04 g citric acid, 1.2 g OSA, 120 mL acetonitrile, $H_20$ up to 2L, pH=6.0 using 50% NaOH solution). The mobile phase was degassed for 10 minutes using the Bransonic® Ultrasonic Bath before being loaded into the HPLC machine.

The standards for HPLC were made by generating master stocks of 100 mM dopamine and 10 mM serotonin diluted in MilliQ $H_2O$, which were kept at 4°C. The day of HPLC analysis, the master stocks were diluted to produce standards ranging from 5-100 nM for 5-HT and 5-1000 nM for dopamine. Sample concentrations of dopamine (Sigma-Aldrich, Cat#H8502) and serotonin (Sigma-Aldrich, Cat#H7752) were calculated based on standards run in the same batch. Standards were compared to standards run on other days to assess for overall performance.

### RNA expression analysis

Flies were raised at 29°C until 5-7 dpe, then heads were collected by cutting them off with a razor blade. Twenty heads were collected per sample with a mixture of males and females and placed directly on dry ice. Samples were kept on dry ice or at -80°C until RNA isolation. For RNA isolation samples were homogenized in 200µL of TRIzol®. Then 800µL of TRIzol® was added and the samples were mixed by pipetting. The samples incubated for 5 minutes at room temperature and were then spun down at 12,000 rpm for 10 minutes at 4°C. The supernatant was removed and 200µL of chloroform was added. The samples were mixed by shaking and then incubated at room temperature for 3 minutes. Then, they were spun at 10,000 rpm for 15 minutes at 4°C, and the aqueous layer was collected and placed in a new tube. 500µL of isopropanol was added and the samples were left to incubate for 10 minutes at room temperature. The samples were spun at 10,000 rpm for 10 minutes at 4°C and the supernatant was removed. The pellet was washed with 1mL of 75% EtOH and centrifuged at 5,000 rpm for 5 minutes. All EtOH was removed and the pellet dried at room temperature for 10 minutes. Then, the pellet was dissolved in 100µL of $H_2O$. RNA content and quality was measured using DeNovix DS-11 Fx spectrophotometer/fluorometer. cDNA reverse transcription was performed according to the iScript™ reverse transcriptase Bio-Rad kit and 2 µL of RNA sample was added in a 10 µL reaction for both the reverse and no reverse transcriptase reaction. Samples were measured for content and quality and then they were diluted to 100ng/µL. qPCR was performed using Bio-Rad iQ SYBR Green Supermix and measured on Bio-Rad CFX96™ Real-Time System. The TH primers used Forward: 5'-ATGTTCGCCATCAAGAAATCCT-3' and Reverse: 5'-GGGTCTCGAAACGGGCATC-3', and the control primers were for Rpl32 were Forward: 5'-ATGCTAAGCTGTCGCACAAATG-3' and Reverse: 5'-GTTCGATCCGTAACCGATGT-3'. For each sample, two technical replicates were run for the reverse transcriptase reaction, and the final quantity was based on the average of the two. If the no reverse transcriptase reaction produced a product that was a Cq within ten, the sample was discarded.

### DAN and TH Quantification

Samples were stained and imaged like the brain expression analysis, except the primarily antibody solution included an Elav antibody [anti-Tyrosine Hydroxylase 1:500, PelFreez Biologicals, rabbit, P40101; anti-Elav 1:100, Developmental Studies Hybridoma Bank, rat, 7E8A10 in solution (5% Normal Donkey Serum, 0.1% $NaN_3$ in 0.5% PBST)]. Confocal imaging was performed as described above. Once the samples were imaged, anterior, posterior, and whole brain projections were generated that included all fluorescent signals from the anterior (PAL and PAM clusters) and posterior (PPL1, PPM2, PPM3, PPL2ab) neuron clusters of interest. The number of neurons per cluster (excluding the PAM cluster) were counted for each sample. PPM2 and PPM3 clusters were counted together. For TH protein quantification samples were assessed using ImageJ. A boundary was drawn around each individual cluster and quantified. Then, the local background for that cluster was subtracted from the cluster to give the fluorescence for the given sample. Each hemisphere was treated as a separate sample.

### Statistical Analysis

Statistical analysis was performed using Graphpad Prism 10. Unless otherwise stated, all data was subjected to a ROUT outlier test, where all outliers were removed, and then a one-way ANOVA was performed. Each of the experimental samples were compared to the control for the given samples. In the cases where there were only two samples (i.e. control and experimental), a t-test was performed.

For the HPLC screen on heads, the samples were normalized to the controls run on that day. The TRiP collection was normalized to *UAS-mCh RNAi* control and the NIG collection was normalized to the *UAS-lacZ RNAi* control.

## Supplemental Information

Document S1. Figures S1-S10

Table S2. Excel file containing data too large to fit in a PDF Table S1-S5

**S1 Fig. GO term enrichment analysis performed on the pigmentation screen hits.**

**S2 Fig. Comparison between genes identified as hits from our pigmentation screen to those tested in the original thorax screen (Mummery-Widmer et al., 2009).**

**S3 Fig. Comparison of dopamine levels between lacZ RNAi and mCh RNAi lines in the fly head.**

**S4 Fig. Validation of recombined GAL4 lines used for dopamine measurement in the brain.**

**S5 Fig. Brain data for prioritized genes with no change in brain dopamine.**

**S6 Fig. Additional clusters of TH positive neurons upon *mask* knockdown show trend or little effect.**

**S7 Fig. Sleep behavior analysis upon TH GAL4 driven TH knockdown.**

**S8 Fig. Additional sleep analysis from TH-GAL4 driven *mask* knockdown.** (

**S9 Fig. Sleep behavior analysis of *clueless* knockdown highlights a different pathway than mask.**

**S10 Fig. Comparison of RNAi lines from the pigmentation screen**

**S11 Fig. Expression analysis for other prioritized candidate genes that did not show any significant change in total head dopamine**.

**S1 Table. Pigmentation screen results by RNAi line**

**S2 Table. Drosophila Activity Monitor results by RNAi line**

**S3 Table. HPLC results by RNAi line**

**S4 Table. Prioritized gene list for HPLC**

**S5 Table. All RNAi lines used with stock center information**
